# Comparison between Long-Menu and Open-Ended Questions in computerized medical assessments. A randomized controlled trial

**DOI:** 10.1186/1472-6920-6-50

**Published:** 2006-10-10

**Authors:** Thomas Rotthoff, Thomas Baehring, Hans-Dieter Dicken, Urte Fahron, Bernd Richter, Martin R Fischer, Werner A Scherbaum

**Affiliations:** 1 Department of Endocrinology, Diabetes and Rheumatology University Hospital, Moorenstr. 5, 40225 Duesseldorf, Germany; 2 German Diabetes Centre at Duesseldorf University, Auf'm Hennekamp, 40225 Duesseldorf, Germany; 3 Central Institute of Clinical Chemistry and Laboratory Medicine, University Hospital Duesseldorf, Moorenstr. 5, 40225 Duesseldorf, Germany; 4 University of Munich Hospital, Medizinische Klinik Innenstadt, Schwerpunkt Medizindidaktik, Ziemssenstr. 1, 80336 Munich, Germany

## Abstract

**Background:**

Long-menu questions (LMQs) are viewed as an alternative method for answering open-ended questions (OEQs) in computerized assessment. So far this question type and its influence on examination scores have not been studied sufficiently. However, the increasing use of computerized assessments will also lead to an increasing use of this question type.

Using a summative online key feature (KF) examination we evaluated whether LMQs can be compared with OEQs in regard to the level of difficulty, performance and response times. We also evaluated the content for its suitability for LMQs.

**Methods:**

We randomized 146 fourth year medical students into two groups. For the purpose of this study we created 7 peer-reviewed KF-cases with a total of 25 questions. All questions had the same content in both groups, but nine questions had a different answer type. Group A answered 9 questions with an LM type, group B with an OE type. In addition to the LM answer, group A could give an OE answer if the appropriate answer was not included in the list.

**Results:**

The average number of correct answers for LMQs and OEQs showed no significant difference (p = 0.93). Among all 630 LM answers only one correct term (0.32%) was not included in the list of answers. The response time for LMQs did not significantly differ from that of OEQs (p = 0.65).

**Conclusion:**

LMQs and OEQs do not differ significantly. Compared to standard multiple-choice questions (MCQs), the response time for LMQs and OEQs is longer. This is probably due to the fact that they require active problem solving skills and more practice. LMQs correspond more suitable to Short answer questions (SAQ) then to OEQ and should only be used when the answers can be clearly phrased, using only a few, precise synonyms.

LMQs can decrease cueing effects and significantly simplify the scoring in computerized assessment.

## Background

The curricular requirements for medical students have changed worldwide during recent years. Future physicians must be better prepared for a changing health care environment, which demands improved decision-making capabilities [1]. In our experience students generally do not lack the fund of knowledge, but rather have difficulties in developing strategies for problem solving. As medical school training has changed, so have the requirements for assessments of knowledge, skills and decision-making capabilities. During recent years different styles of questioning have been developed for written assessments to test the cognitive abilities of the students rather than just the memorized facts.

The validity of an assessment can be increased by focusing more on real life situations and medical practice. As a consequence more questions are set in a clinical context [2]. One of types of questions is based on clinical cases. They allow assessment of clinical decision-making in a key-feature (KF) form as well as the assessment of theoretical knowledge. A key feature is defined as a critical step in the resolution of a problem [3][4]. For such chronologically organized case-based assessments computerized tests provide methodological advantages. Furthermore, computerized assessments allow the use of different question and answer types. In addition to multiple-choice (MC) and open-ended questions (OEQs), short-menu (SM) and long-menu questions (LMQs) can be used. LMQs are open-ended questions in which students have to fill in a specific term but do not have to write an analysis or summary as with essay questions.

LMQs were developed to avoid cueing effects of MCQs. Veloski et al. (1993) used LMQs, applying a long alphabetical list of possible answers [5]. Despite the expected decrease in the cueing effect, the method was still highly time-consuming. They were not very practical and caused transmission errors. The computer-based form [6] of the LMQ type was used for the first time by Schuwirth et al. (1996). Students entered their answer into a dialog box and the computer compared it with a list of more than 2500 possible answers. A selection of answers was displayed to the students who then selected an answer. Schuwirth et al. compared the use of LMQs with OEQs for the first time. Students answered questions initially as OEQs and thereafter in the LM format. In this study the two groups were not independent of each other though.

Fischer et al. (2005) used LMQs as an alternative to OEQs [7] in a study to validate key features during an online assessment. They showed that an electronic key feature assessment is feasible and can produce reliable assessment results. However, the performance of an LM format was not evaluated in this study. The increased use of computerized assessments will most likely also lead to an increased use of this question format.

In our study we used a summative online KF examination to evaluate differences in level of difficulty, performance and response times between LMQs and OEQs of identical content.

## Methods

### Design

The assessment was carried out at the end of the winter term in February 2005. Before the examination date all students had the opportunity to familiarize themselves with the system through voluntary exercises with cases in haematology and endocrinology, which were included in a computerized assessment programme (CASUS™). A total of 133 students practised the exercise. The cases contained OEQs but no LMQs. This means that the students used the LM format for the first time during the examination. Five days prior to the assessment all students were invited to an information session about the new assessment format and the study design. The assessment was a regular part of their studies and gave them a chance to get bonus points for the final grade in Internal Medicine.

**Figure 1:**
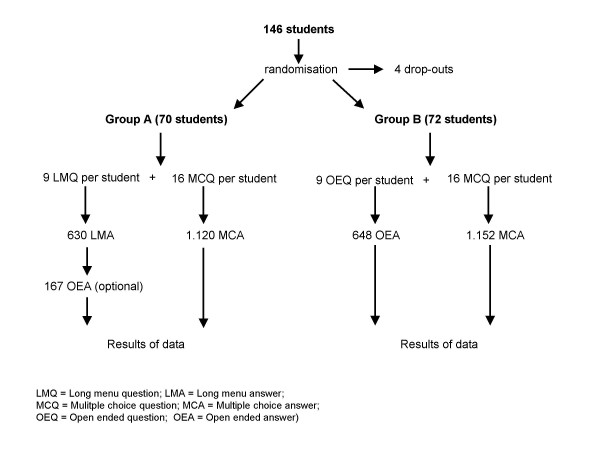
Design of the study.

The students were randomized into two groups, A and B, by use of a computer algorithm (Figure 1 - design of the study). A person not belonging to the study team assured concealment of allocation. All questions used in the assessment were identical for both groups, but nine of them were different in the answer format. Group A answered nine questions using an LM option, while group B used an OE option. The remaining 16 questions were identical for both groups. After entering the LM answer, students in group A were given the chance to type their answer term into a additional text cell, immediately following the LM window. This additional text cell is not a usual part of the LM questions but was included in our study to test the handling of LM answers, in case the answer was not included in the list or the LM list would not function properly. However, it allowed all students the opportunity to enter a verbal text and therefore provided equal chances in answering the questions in this real examination. Because the students were given this opportunity they had no objections to the study. Like in group B, the OE cells were evaluated manually after the assessment.

### Long menu system

As soon as the answer was typed into the appropriate dialogue box, the computer compared it with an alphabetical list and the terms were shown in a pop-up scroll down menu. For example, if the answer was ketoacidosis and the letters 'keto' were entered, the computer would display the terms ketoacidosis, ketoconazole, ketolides, ketotifen from the LM list. As the entire text was typed in, the computer selected the appropriate term, which then had to be confirmed. Because distractors were included in the LM list, wrong answers were also displayed.

Our LM list included terms from Internal Medicine, covering diagnosis, diagnostic procedures and therapy. It used more than 500 terms out of a body of 8000 MC questions from the second State Medical Exam. When answering the questions, students were able to change their answers until they had confirmed them.

### Generation of a LMQ

In the following, the generation of a LMQ is described by an example:

One vignette describes a patient with anaemia who also suffers from Crohn's disease and had a resection of the distal ileum secondary to strictures. The student is shown a picture of a face with a fissure at the angle of the mouth.

What type of anaemia is this most likely?

Table 1 shows the author's view of the answer key. It defines the correct answer. Possible synonyms are separated by the author through vertical lines.

This example shows the determination of answers and distractors and the large number of possible distractors which contribute to an expansion of the LM list. The more complete the LM list becomes for individual topics, the easier it is to develop questions, because all possible distractors are already included in the list. For example, if in another question the blood smear of a patient with sickle cell anaemia is shown and the students are asked for the correct diagnosis, all distractors for anaemia would already be given in the question to B-12 deficiency anaemia. The author would then only have to define the correct answer, because the entire LM list would serve as a distractor.

### Participants

Out of 146 randomized fourth year medical students, 142 students (66 male and 76 female) participated in the study. Four students were not present on the day of the assessment.

### Testing material

Seven peer-reviewed hypothetical clinical cases were used for this study. They included a total of 25 questions from endocrinology and haematology. Each case contained three to five questions which focused on clinical decision making. The cases were prepared in a modified key feature format. Out of 25 questions, five dealt with achieving to a diagnosis, seven with the diagnostic procedure, eight with therapeutic decisions and five questions assessed the pathophysiological reasoning .

Altogether 16 questions were MCQs and nine questions were LMQs (Group A) and OEQs (Group B). The web-based system CASUS™ [8][9] was used for the examination in the University Computer Centre.

### Scoring

Answers to MCQs and LMQs were evaluated electronically. Students received one point for each item. Questions with two correct answers were only counted when both answers were marked properly (two MCQs and three LMQs). All OEQs were evaluated by two examiners, using a previously defined answering key. The final score was a summary of the percentage of correct answers. Response time for a question was measured in seconds. To pass the exam, 60% of the answers had to be correct.

### Analysis

Based on the null hypothesis of equal means of correctly answered questions out of a total of nine questions of the LM and OE type, we tested for differences. The α-type error was set at *p *= 0.05. Differences between individual items were analyzed additionally. Chi-square and Fisher's exact tests were carried out using StatsDirect Statistical Software (Version 2.2.6). Response times for LMQs- and OEQs were evaluated with unpaired t-tests.

## Results

### 1. Comparison of exam results by groups

#### Comparison of Long-Menu Questions (LMQs) and Open-Ended Questions (OEQs)

Table 2 shows the results of each question by randomised groups. There was no significant difference between the means of correct LM and OE answers (*p *= 0,92). The mean percentage of correct answers for LMQs was 73.3% (*SD *16.7) and for OEQs 73.5% (*SD *19.2). Apart from item no. 3, there was no significant difference between individual items. This question showed 50% correct answers in the LM part and 66.7% in the OE part (*p *= 0.0439). In this item it was asked for the two main therapeutic interventions in case of a decompensated ketoacidosis.

Within the MC questions, the mean percentage of correct answers was 67.4% (*SD *25.2) in group A and 69.8% (*SD *25.3) in group B, demonstrating no significant difference.

#### Response time

On average, response time for LM as well as OEQs was longer than MCQs, despite the fact that MCQs on average were more difficult to solve. Calculation of the LM response time was based on the LM answer and not on possible entries into the OE text cell. Mean response time for LMQs was 101 seconds (*SD *32), compared with 107 seconds (*SD *31) for OEQs, demonstrating no significant difference (*p *= 0.65).

#### Influence of LMQs on assessment results

With a minimum score of 60% correct answers, 54 out of 70 students (77.1%) in the LM group A (OE text cells were not rated) and 61 out of 72 students (84.7%) of group B passed the assessment. For seven students, manual evaluation of the optional OE text cells resulted in a higher score, which allowed them to finally pass the assessment. Therefore the number of successful LM students increased from 54 to 61 (87.1%).

### 2. Analysis of individual OE Answers in the Long-Menu group

**Figure 2:**
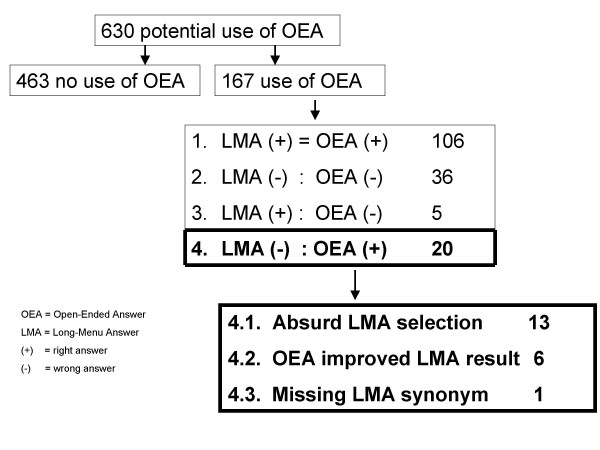
Analysis of the applicability of the long menu format (numbers indicate answers).

Students in the Long-Menu group entered in 167 / 630 cases (26.5%) an answer into the OE text cell. (Figure 2).

In sixty-four percent of cases the answer in the LM was identical to the answer in the OE text cell. Thirty-six answers were wrong both in the LM and in the OE cell. Only 20 (3.2%) out of 630 possible answers in group A were answered incorrectly in the LM cell but correctly in the OE-text cell. Further examination showed implausible terms in 13 out of these 20 LM answers, for example, ketoacidosis would have been the correct answer, but pericardial tamponade was selected in the long menu. The 20 correct answers in the OE text cell were, apart from one single term, all included in the long menu list and therefore previously defined as correct by the authors. The single term which was not included in the list (dextrose) equals a proportion of 0.2% of all LM answers. In six cases answers in the OE text cell were more precise, so that they could be rated as correct. In five cases there was a correct answer in the LM cell, but an incorrect one in the OE text cell. In these cases the answer was rated as correct.

## Discussion

Our study showed that computer based LMQs are feasible and do not differ significantly from OEQs. Only one question was answered significantly more often incorrectly in the LM group compared to the OE group. This is probably due to chance, because most of the students had also inserted an incorrect term into the optional OE text cell and therefore did not improve their results.

### Difficulties in the development of LMQs

It became obvious that clear and unambiguous phrasing of answers is of paramount importance in the preparation of LMQs. Ideally, only a few synonyms or different phrases should be provided for an answer and optimally only one single answer term. Each correct answer and its synonyms have to be defined exactly by the author.

The difficulty for the author in developing LMQs is to anticipate all possible answering terms. With complex answers it can become more difficult to consider all possible synonyms and an automated analysis becomes more erroneous. For this reason the phrasing of the questions is essential, because it allows to channel the answers into a certain direction. This is demonstrated in the following sample case with two different versions of the same question. A 23 year old patient with symptoms of an acute ketoacidosis.

### Version A

#### Which two therapeutic interventions are indicated most urgently in this situation?

The phrasing of the question allows several possible correct answers as shown in table 3:

By rephrasing the question the number of possible answers can be reduced.

### Version B

#### What should be given most urgently in this situation?

Now the syntax of the terms marked bold in table 4 does not fit anymore and the number of possible answers is reduced, which could avoid mistakes in the analysis.

In the ideal case scenario only a few synonyms or spellings for an answer should exist, which would facilitate the use of key words. If appropriate synonyms are not considered by the author, the students cannot find them in the LM list. In the study by Fischer et al. [7] exactly this fact was criticized by some students. Alternatively, the appropriate synonyms could have been included in the LM list, but the author did not define them as a correct answer.

These comments imply that the LM format is not appropriate for open-ended questions with whole sentence entries, because it is impossible to match them alphabetically with the LM list. The LMQs correspond thereby more suitable to short answer questions.

Although our list included fewer terms than the one used by Schuwirth et al. [6], the overall length of the list seems to be of minor importance: Only 20 (3.2%) out of 630 possible LM answers in group A were incorrect in the LM cell, with the correct answer subsequently inserted in the OE text cell. Apart from one term (dextrose), all terms written down in the OE text cell were included in the LM list. Most likely these problems were caused by slips and handling errors in using the LM list. Indeed, most errors happened with the first LM question (item 1). The students were shown the selected answer term again before using the send' button. This makes a technical or system error unlikely.

Some of the mistakes may be explained by the fact that the students used this answer format for the first time. In 36 cases an incorrect answer was inserted in the LM cell as well as in the subsequent OE text cell. This means that the incorrect answers did not result from an incomplete LM list but from difficulties in answering the questions correctly. After a manual evaluation of all OEQs seven students could improve their score and finally pass the exam. In order to minimize mistakes that are caused by slips and handling errors, students should be given the opportunity to gain some experience with the LM format during the term. The problem of a small size LM list, which has been described by Schuwirth and Fischer, seems to be of little importance [6][7]. Important, however, is the fact that the wording of the LMQs must be specific, the answering terms should be unambiguous and the number of synonyms limited.

In our study as well as in the study by Fisher et al.[7], on average LMQs and OEQs were easier to solve than MC questions, although response time was longer for LMQs and OEQs [7]. The fact that for certain items more answers were correct and had to be marked to score a point resulted in a higher degree of difficulty. This applied to LM and OEQs alike (item 3 and 10) as well as to MC questions (item 2 and 9). It remains unclear whether the content of LMQs or OEQs or the LM or OE format alone resulted in a lower degree of difficulty. In our study, most LMQs/OEQs were used to evaluate knowledge related to diagnosis (e.g. ketoacidosis, pernicious anaemia, hypoglycemia), diagnostic procedure (e.g. potassium test) and therapy (e.g. metformin, insulin) in the key feature cases. Thereby, we assessed relevant clinical decision making abilities, which are considered important for undergraduate medical students [10]. Within the MCQs we also asked for the pathophysiological reasoning (e.g. interpretation of a blood gas analysis in case of decompensated diabetes mellitus type 1 or control after initialisation of an insulin therapy). However, only item Nr. 24 (explanation of the mechanism for developing vitamin B 12 deficiency anaemia) had a high degree of difficulty (0.4).

The findings of our study are limited by the restricted number of LMQs, although they were tested on a large number of participants. The small number of LMQs is due to the fact that in a real exam situation an exclusive use of LMQs does not appear to be reasonable. In addition, choosing the questions exclusively from the subspecialties of endocrinology and haematology limits our findings. However, because we were asking primarily for diagnoses, diagnostic procedures or therapies, the use of LMQs seems to be independent of a specialty.

A further point of criticism of the study is the provision of an OE comment box with the LMQs. Candidates might think that the answer was missing, but the presence of this option may have influenced the candidates to think or react differently.

In this study we used an LM list which included different areas, like diagnoses and diagnostic and therapeutic procedures. Each case study author had the opportunity to use LMQs and therefore choose an arbitrary number of answers and distractors. This makes the system prone to errors and warrants a central management of the LM list to avoid double entries, orthographic mistakes and general chaos. Different LM lists for diagnoses, diagnostic procedures and therapies should be considered to achieve a better overview and manageability. The ICD 10, for example, could serve as a list of diagnoses. In the future the scrollable pop-up menu could be omitted. The students are meant to develop an answer spontaneously and enter it into the LM answer field to avoid searching for an appropriate answer in the LM list. In this respect the format differs from classical MCQ and extended-matching MCQ. Furthermore, the terms displayed in an alphabetical order in the pop-up menu do not necessarily constitute suitable distractors. During recent years it has been shown that not the answer format itself, but the stimulus set by the question (e.g. integration of the question into a medical context) influences the results [11][12]. Each question and answer type has its own advantages and disadvantages. The decisive factor for a selection is the purpose of the question and answer. An MCQ, for example, doesn't seem to be suitable when students are asked to spontaneously generate a diagnosis.

Within our study, LM and OEQs were used in form of short-answer questions. Students were asked for clearly defined terms instead of lengthy analyses. In the literature a transformation of OEQs into short answer questions is generally viewed critically because of the large resources needed and the possibility to replace them by MCQs [11]. However, using computerized analysis with an underlying LM list saves resources and facilitates the evaluation. This is especially useful for specific tasks, such as generating a diagnosis or planning diagnostic and therapeutic procedures. This method may also be used in other specialties. After an LM list has been generated, it neither requires much time nor resources to add new questions. Easy access to a computer based assessment format allows university-wide use of the LM list. This means individual faculties do not have to create separate lists.

Authenticity, clinical decision-making and validity of assessments might be increased when answers of the LM type are used within a key feature assessment. They combine a stimulus for clinical decision-making with the opportunity of generating a spontaneous answer. This procedure appears to be very similar to clinical reality.

The decision about the appropriate answer format should be based on the content of the question. Psychometric measurements should not be overvalued when the answer format assesses important aspects and increases validity [13].

## Conclusion

LMQs do not seem to be more difficult, although the answer terms generated by the author have to correlate with those of the students. Compared with typical MCQs, LMQs as well as OEQs need a longer response time. This might result from a different stimulus or lack of experience in using this format. Although a subsequent manual evaluation improved the overall assessment scores, we found that slips and handling errors were probably the main reasons. This problem could be reduced if students would use this question format more frequently. However, the length of the LM list was not a significant problem. LMQs correspond more suitable to short-answer questions then to OEQ and should only be used when the answers can be clearly phrased, using a few, precise synonyms.

## Competing interests

The author(s) declare that they have no competing interests.

## Authors' contributions

TR and TB had the idea of the project and were responsible for general concept and study design. They accept full responsibility for the finished article, had access to all data, and controlled the decision to publish. MRF supported framing of the study design. TR and UF constructed the cases for examination. TB organized the technical implementation. HDD evaluated the results and edited the text. TR performed the literature search and BR did and interpreted the statistical analysis.

TR, TB, HDD and UF wrote the article and WAS was critical involved in the establishment of the case-based e-assessment. WAS, BR and MRF revised the article critically. All authors reviewed the final manuscript.

## Pre-publication history

The pre-publication history for this paper can be accessed here:



## Figures and Tables

**Table 1:** Example for the construction of an LMQ by the author

Answer 1:	**Answer**	Megaloblastic anaemia | Vitamin B12 deficiency anaemia | B-12 deficiency anaemia | folic acid deficiency anaemia
Answer 2:	Distractor	Pernicious anaemia
Answer 3:	Distractor	Iron deficiency anaemia
Answer 4:	Distractor	Infectious anaemia
Answer 5:	Distractor	Tumor anaemia
Answer 6:	Distractor	Autoimmune haemolytic anaemia | AIHA
Answer 7:	Distractor	Beta thalassaemia | β-Thalassaemia
Answer 8:	Distractor	Glucose-6-phosphatedehydrogenase deficiency
Answer 9:	Distractor	Sickle cell anaemia | Sickle cell disease
Answer 10:	Distractor	Pyruvate kinase deficiency
Answer 11:	Distractor	Aplastic anaemia
Answer 12:	Distractor	Autoimmune haemolysis

**Table 2:** Results of Multiple-Choice- (MC), Long-Menu- (LM) and Open-Ended (OE) questions by randomised groups

**Item No**	**group A**			**group B**		
	
	**LM**	**MC**	**mean time (s) to answer item**	**OE**	**MC**	**mean time (s) to answer item**
				
	% of correct answer			% of correct answer		
**1**	75,7		129,6	65,3		95,6
**2**		22,9	123,4		25,0	137,9
**3**	50,0		126,8	66,7		107,6
**4**		68,6	82,3		79,2	83,2
**5**		84,3	47,7		98,6	47,1
**6**		85,7	48,1		87,5	52,9
**7**	82,9		59,0	84,7		77,5
**8**	95,7		64,6	97,2		71,4
**9**		18,6	105,4		9,7	90,6
**10**	48,6		121,3	40,3		99,5
**11**	84,3		74,7	93,1		93,2
**12**		51,4	85,3		55,6	90,7
**13**		50,0	107,9		48,6	121,2
**14**		82,9	85,8		76,4	101,1
**15**		50,0	67,7		70,8	74,9
**16**	62,9		139,1	55,6		122,7
**17**		82,9	89,0		86,1	104,7
**18**		91,0	57,6		91,7	79,5
**19**	71,4		117,8	68,1		173,8
**20**		82,9	75,5		73,6	121,8
**21**		97,1	39,0		90,3	51,9
**22**	88,6		71,8	90,3		125,2
**23**		92,9	28,2		86,1	35,0
**24**		40,0	40,6		51,4	56,3
**25**		77,1	61,6		86,1	57,2

**Table 3:** Answer opportunities depending on verbalization of the question

Answer 1:	Answer	Volume substitution | fluids | NaCl | sodium chloride solution | p.o. intake |
Answer 2:	Answer	Insulin
Answer 3:	Distractor	Bicarbonate | buffering | correction of acidosis
Answer 4:	Distractor	Catecholamines

**Table 4:** Answer opportunities by rephrasing the same question

Answer 1:	Answer	**Volume substitution **| fluids | NaCl | sodium chloride solution | **p.o. intake **|
Answer 2:	Answer	Insulin
Answer 3:	Distractor	Bicarbonate | **buffering **| **correction of acidosis**
Answer 4:	Distractor	Catecholamines
